# 2,6-Bis(2-methyl-1,3-diazinan-2-yl)pyridine

**DOI:** 10.1107/S1600536810050063

**Published:** 2010-12-08

**Authors:** Quoc-Cuong Ton, Michael Bolte

**Affiliations:** a Institut für Organische Chemie und Chemische Biologie, Goethe-Universität Frankfurt, Max-von-Laue-Strasse 7, 60438 Frankfurt/Main, Germany; b Institut für Anorganische und Analytische Chemie, Goethe-Universität Frankfurt, Max-von-Laue-Strasse 7, 60438 Frankfurt/Main, Germany

## Abstract

The title compound, C_15_H_25_N_5_, is an aminalization product between 2,6-diacetyl­pyridine and 1,3-diamino­propane. It crystallizes with two independent mol­ecules in the asymmetric unit with different conformations. In the first mol­ecule, the methyl groups are *cis* oriented with respect to the pyridine ring [N—C—C—C torsion angles = 72.5 (1) and 80.3 (1)°], while they are *trans* oriented in the second mol­ecule [N—C—C—C torsion angles = 82.6 (1) and −90.8 (1)°]. Each of the two mol­ecules forms centrosymmetric dimers held together by N—H⋯N hydrogen bonds, thus forming *R*_2_^2^(16) rings. The two dimers are inter­linked by additional N—H⋯N bonds into *R*_4_^4^(14) rings, building chains along the *a* axis. These patterns influence the orientation (either equatorial or axial) of the N—H bonds.

## Related literature

For 2,6-diacetyl­pyridine, see: Burnet *et al.* (2003 ▶) and for 1,3-diamino­propane, see: Thalladi *et al.* (2000 ▶).
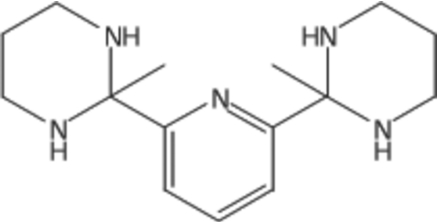


## Experimental

### 

#### Crystal data

C_15_H_25_N_5_
*M* *_r_* = 275.40
Monoclinic, 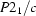

*a* = 18.715 (4) Å
*b* = 7.512 (2) Å
*c* = 22.730 (5) Å
β = 102.07 (3)°
*V* = 3124.9 (13) Å^3^
*Z* = 8
Mo *K*α radiation
μ = 0.07 mm^−1^
*T* = 173 K
0.40 × 0.32 × 0.30 mm

#### Data collection

Stoe IPDS II two-circle diffractometer
41522 measured reflections
5758 independent reflections
4924 reflections with *I* > 2σ(*I*)
*R*_int_ = 0.050

#### Refinement

*R*[*F* ^2^ > 2σ(*F* ^2^)] = 0.036
*wR*(*F* ^2^) = 0.098
*S* = 1.35
5758 reflections
394 parameters
H atoms treated by a mixture of independent and constrained refinement
Δρ_max_ = 0.28 e Å^−3^
Δρ_min_ = −0.18 e Å^−3^

### 

Data collection: *X-AREA* (Stoe & Cie, 2001 ▶); cell refinement: *X-AREA*; data reduction: *X-AREA*; program(s) used to solve structure: *SHELXS97* (Sheldrick, 2008 ▶); program(s) used to refine structure: *SHELXL97* (Sheldrick, 2008 ▶); molecular graphics: *XP* (Sheldrick, 2008 ▶); software used to prepare material for publication: *publCIF* (Westrip, 2010 ▶).

## Supplementary Material

Crystal structure: contains datablocks I, global. DOI: 10.1107/S1600536810050063/ng5081sup1.cif

Structure factors: contains datablocks I. DOI: 10.1107/S1600536810050063/ng5081Isup2.hkl

Additional supplementary materials:  crystallographic information; 3D view; checkCIF report

## Figures and Tables

**Table 1:** Hydrogen-bond geometry (Å, °)

*D*—H⋯*A*	*D*—H	H⋯*A*	*D*⋯*A*	*D*—H⋯*A*
N2—H2*N*⋯N5^i^	0.893 (16)	2.596 (15)	3.3984 (16)	149.9 (12)
N4—H4*N*⋯N4′^ii^	0.908 (16)	2.623 (16)	3.4716 (16)	155.8 (13)
N3′—H3′*N*⋯N5′^iii^	0.873 (15)	2.418 (15)	3.2662 (17)	164.2 (12)

Symmetry codes: (i) 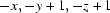; (ii) 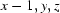; (iii) 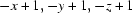.

**Fig. 1.**
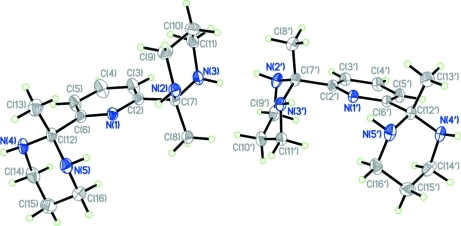
A perspective view of (I), showing the atom-numbering scheme. Displacement ellipsoids are drawn at the 50% probability level and H atoms are shown as small spheres of arbitrary radii.

**Fig. 2.**
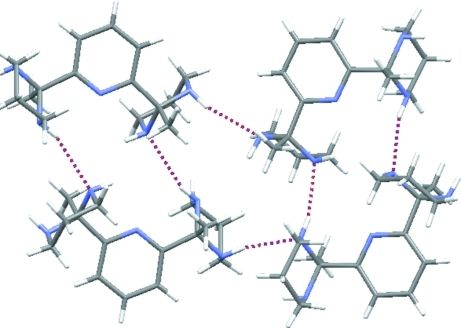
A partial packing diagram for (I). N—H···N hydrogen bonds are shown as dashed lines.

**Fig. 3.**
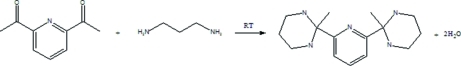
Reaction scheme between 2,6-diacetylpyridine and 1,3-diaminopropane.

## supplementary crystallographic information

### Comment

The aim of this investigation was to cocrystallize 2,6-diacetylpyridine (Burnet *et al.*, 2003) with 1,3-diaminopropane (Thalladi *et al.*, 2000). Unfortunately crystals of the title compound were obtained due to an aminalization reaction between the starting compounds (Fig. 2).

### Experimental

The starting compounds were purchased from Aldrich and Lancaster and utilized for a cocrystallization experiment without purification. 2,6-diacetylpyridine (10 mg) was added to an excess of 1,3-diaminopropane (0.8 ml). The mixture in a flask was set aside at room temperature. After several months colourless crystals were obtained.

### Refinement

H atoms bonded to C were refined with fixed individual displacement parameters [U(H) = 1.2 *U*_eq_(C)] using a riding model with C_aromatic_—H = 0.95 Å, C_methylene_—H = 0.99 Å, or C_tertiary_—H = 0.98 Å, respectively. H atoms bonded to N were freely refined.

### Crystal data

**Table:**

C_15_H_25_N_5_	*F*(000) = 1200
*M**_r_* = 275.40	*D*_x_ = 1.171 Mg m^−^^3^
Monoclinic, *P*2_1_/*c*	Mo *K*α radiation, λ = 0.71073 Å
Hall symbol: -P 2ybc	Cell parameters from 38746 reflections
*a* = 18.715 (4) Å	θ = 3.3–25.7°
*b* = 7.512 (2) Å	µ = 0.07 mm^−^^1^
*c* = 22.730 (5) Å	*T* = 173 K
β = 102.07 (3)°	Block, colourless
*V* = 3124.9 (13) Å^3^	0.40 × 0.32 × 0.30 mm
*Z* = 8	

### Data collection

**Table:**

Stoe IPDS II two-circle diffractometer	4924 reflections with *I* > 2σ(*I*)
Radiation source: fine-focus sealed tube	*R*_int_ = 0.050
graphite	θ_max_ = 25.5°, θ_min_ = 3.3°
ω scans	*h* = −22→22
41522 measured reflections	*k* = −9→9
5758 independent reflections	*l* = −27→27

### Refinement

**Table:**

Refinement on *F*^2^	Secondary atom site location: difference Fourier map
Least-squares matrix: full	Hydrogen site location: inferred from neighbouring sites
*R*[*F*^2^ > 2σ(*F*^2^)] = 0.036	H atoms treated by a mixture of independent and constrained refinement
*wR*(*F*^2^) = 0.098	*w* = 1/[σ^2^(*F*_o_^2^) + (0.0513*P*)^2^] where *P* = (*F*_o_^2^ + 2*F*_c_^2^)/3
*S* = 1.35	(Δ/σ)_max_ < 0.001
5758 reflections	Δρ_max_ = 0.28 e Å^−^^3^
394 parameters	Δρ_min_ = −0.18 e Å^−^^3^
0 restraints	Extinction correction: *SHELXL97* (Sheldrick, 2008), Fc^*^=kFc[1+0.001xFc^2^λ^3^/sin(2θ)]^-1/4^
Primary atom site location: structure-invariant direct methods	Extinction coefficient: 0.0182 (13)

### Special details

**Table:**

Geometry. All e.s.d.'s (except the e.s.d. in the dihedral angle between two l.s. planes) are estimated using the full covariance matrix. The cell e.s.d.'s are taken into account individually in the estimation of e.s.d.'s in distances, angles and torsion angles; correlations between e.s.d.'s in cell parameters are only used when they are defined by crystal symmetry. An approximate (isotropic) treatment of cell e.s.d.'s is used for estimating e.s.d.'s involving l.s. planes.
Refinement. Refinement of *F*^2^ against ALL reflections. The weighted *R*-factor *wR* and goodness of fit *S* are based on *F*^2^, conventional *R*-factors *R* are based on *F*, with *F* set to zero for negative *F*^2^. The threshold expression of *F*^2^ > σ(*F*^2^) is used only for calculating *R*-factors(gt) *etc*. and is not relevant to the choice of reflections for refinement. *R*-factors based on *F*^2^ are statistically about twice as large as those based on *F*, and *R*- factors based on ALL data will be even larger.

### Fractional atomic coordinates and isotropic or equivalent isotropic displacement parameters (Å^2^)

**Table:**

	*x*	*y*	*z*	*U*_iso_*/*U*_eq_	
N1	0.01315 (5)	0.64048 (12)	0.38741 (4)	0.0208 (2)	
C2	0.06102 (6)	0.70659 (15)	0.35629 (5)	0.0219 (2)	
C3	0.03859 (7)	0.78073 (19)	0.29935 (6)	0.0337 (3)	
H3	0.0733	0.8227	0.2775	0.040*	
C4	−0.03613 (7)	0.7920 (2)	0.27508 (6)	0.0419 (4)	
H4	−0.0530	0.8449	0.2367	0.050*	
C5	−0.08588 (6)	0.72571 (18)	0.30721 (6)	0.0336 (3)	
H5	−0.1370	0.7337	0.2915	0.040*	
C6	−0.05903 (6)	0.64714 (15)	0.36306 (5)	0.0222 (2)	
C7	0.14237 (5)	0.67781 (15)	0.38678 (5)	0.0205 (2)	
C8	0.15860 (6)	0.47996 (15)	0.38048 (5)	0.0262 (3)	
H8A	0.1275	0.4091	0.4013	0.039*	
H8B	0.2101	0.4565	0.3984	0.039*	
H8C	0.1486	0.4476	0.3378	0.039*	
N2	0.15809 (5)	0.71862 (13)	0.45185 (4)	0.0226 (2)	
H2N	0.1276 (8)	0.658 (2)	0.4698 (6)	0.034 (4)*	
C9	0.15049 (7)	0.90860 (17)	0.46430 (6)	0.0319 (3)	
H9A	0.1608	0.9292	0.5083	0.038*	
H9B	0.0999	0.9482	0.4473	0.038*	
C10	0.20407 (7)	1.01382 (17)	0.43596 (6)	0.0363 (3)	
H10A	0.2548	0.9798	0.4548	0.044*	
H10B	0.1982	1.1428	0.4426	0.044*	
C11	0.18907 (7)	0.97400 (16)	0.36861 (6)	0.0325 (3)	
H11A	0.1409	1.0243	0.3495	0.039*	
H11B	0.2266	1.0338	0.3507	0.039*	
N3	0.18942 (5)	0.78179 (13)	0.35523 (4)	0.0255 (2)	
H3N	0.2350 (8)	0.7418 (18)	0.3682 (6)	0.028 (3)*	
C12	−0.10820 (6)	0.56698 (15)	0.40310 (5)	0.0228 (2)	
C13	−0.11675 (7)	0.70647 (19)	0.45014 (6)	0.0357 (3)	
H13A	−0.0687	0.7351	0.4749	0.054*	
H13B	−0.1483	0.6593	0.4758	0.054*	
H13C	−0.1388	0.8144	0.4299	0.054*	
N4	−0.18088 (5)	0.52752 (14)	0.36689 (5)	0.0267 (2)	
H4N	−0.2100 (9)	0.506 (2)	0.3935 (7)	0.041 (4)*	
C14	−0.18228 (7)	0.37221 (18)	0.32745 (6)	0.0339 (3)	
H14A	−0.1575	0.4035	0.2944	0.041*	
H14B	−0.2337	0.3429	0.3093	0.041*	
C15	−0.14553 (7)	0.20842 (19)	0.35991 (7)	0.0405 (3)	
H15A	−0.1422	0.1137	0.3303	0.049*	
H15B	−0.1750	0.1627	0.3881	0.049*	
C16	−0.06904 (7)	0.25698 (17)	0.39462 (7)	0.0353 (3)	
H16A	−0.0463	0.1529	0.4180	0.042*	
H16B	−0.0380	0.2916	0.3662	0.042*	
N5	−0.07435 (5)	0.40640 (14)	0.43568 (4)	0.0270 (2)	
H5N	−0.0281 (8)	0.4360 (19)	0.4533 (6)	0.033 (4)*	
N1'	0.51250 (5)	0.59589 (12)	0.38384 (4)	0.0199 (2)	
C2'	0.47119 (6)	0.66760 (14)	0.33387 (5)	0.0204 (2)	
C3'	0.49877 (6)	0.70014 (16)	0.28223 (5)	0.0250 (3)	
H3'	0.4691	0.7533	0.2477	0.030*	
C4'	0.57073 (6)	0.65272 (16)	0.28268 (5)	0.0264 (3)	
H4'	0.5907	0.6729	0.2481	0.032*	
C5'	0.61335 (6)	0.57584 (16)	0.33375 (5)	0.0249 (2)	
H5'	0.6624	0.5416	0.3346	0.030*	
C6'	0.58237 (5)	0.55030 (14)	0.38369 (5)	0.0200 (2)	
C7'	0.39231 (6)	0.71995 (15)	0.33837 (5)	0.0220 (2)	
C8'	0.39689 (6)	0.90350 (16)	0.36847 (6)	0.0302 (3)	
H8'1	0.4269	0.8954	0.4092	0.045*	
H8'2	0.3477	0.9438	0.3705	0.045*	
H8'3	0.4190	0.9886	0.3449	0.045*	
N2'	0.34706 (5)	0.73352 (14)	0.27711 (4)	0.0272 (2)	
H2'N	0.3052 (8)	0.7831 (19)	0.2808 (6)	0.033 (4)*	
C9'	0.33042 (6)	0.55788 (17)	0.24825 (5)	0.0288 (3)	
H9'1	0.3758	0.5078	0.2390	0.035*	
H9'2	0.2950	0.5741	0.2097	0.035*	
C10'	0.29904 (6)	0.42461 (17)	0.28693 (5)	0.0291 (3)	
H10C	0.2955	0.3055	0.2679	0.035*	
H10D	0.2494	0.4621	0.2904	0.035*	
C11'	0.34896 (6)	0.41589 (15)	0.34943 (5)	0.0248 (2)	
H11C	0.3271	0.3373	0.3759	0.030*	
H11D	0.3971	0.3664	0.3465	0.030*	
N3'	0.35806 (5)	0.59662 (13)	0.37478 (4)	0.0221 (2)	
H3'N	0.3836 (8)	0.5952 (18)	0.4116 (7)	0.030 (3)*	
C12'	0.62660 (6)	0.47668 (15)	0.44412 (5)	0.0208 (2)	
C13'	0.66464 (7)	0.63378 (17)	0.48080 (6)	0.0335 (3)	
H13D	0.6278	0.7182	0.4887	0.050*	
H13E	0.6969	0.6935	0.4581	0.050*	
H13F	0.6936	0.5902	0.5191	0.050*	
N4'	0.68328 (5)	0.35340 (13)	0.43324 (5)	0.0256 (2)	
H4'N	0.7143 (7)	0.3368 (18)	0.4685 (6)	0.029 (3)*	
C14'	0.65331 (7)	0.18110 (18)	0.40894 (6)	0.0360 (3)	
H14C	0.6943	0.0982	0.4084	0.043*	
H14D	0.6268	0.1983	0.3668	0.043*	
C15'	0.60164 (7)	0.09624 (18)	0.44454 (7)	0.0407 (3)	
H15C	0.5785	−0.0105	0.4231	0.049*	
H15D	0.6295	0.0584	0.4845	0.049*	
C16'	0.54299 (7)	0.22950 (18)	0.45217 (6)	0.0364 (3)	
H16C	0.5118	0.2578	0.4125	0.044*	
H16D	0.5117	0.1777	0.4779	0.044*	
N5'	0.57811 (5)	0.39283 (15)	0.48008 (4)	0.0281 (2)	
H5'N	0.5420 (9)	0.474 (2)	0.4791 (7)	0.045 (4)*	

### Atomic displacement parameters (Å^2^)

**Table:**

	*U*^11^	*U*^22^	*U*^33^	*U*^12^	*U*^13^	*U*^23^
N1	0.0161 (4)	0.0239 (5)	0.0212 (5)	−0.0001 (3)	0.0011 (3)	−0.0006 (4)
C2	0.0188 (5)	0.0242 (6)	0.0218 (6)	−0.0005 (4)	0.0018 (4)	0.0006 (4)
C3	0.0234 (6)	0.0486 (8)	0.0277 (6)	−0.0040 (5)	0.0017 (5)	0.0137 (6)
C4	0.0289 (7)	0.0595 (9)	0.0320 (7)	−0.0031 (6)	−0.0056 (5)	0.0224 (6)
C5	0.0178 (6)	0.0443 (7)	0.0342 (7)	0.0009 (5)	−0.0045 (5)	0.0100 (6)
C6	0.0173 (5)	0.0243 (6)	0.0236 (6)	0.0012 (4)	0.0009 (4)	−0.0018 (4)
C7	0.0159 (5)	0.0249 (6)	0.0201 (5)	−0.0005 (4)	0.0023 (4)	0.0017 (4)
C8	0.0202 (5)	0.0270 (6)	0.0303 (6)	0.0006 (4)	0.0028 (5)	−0.0012 (5)
N2	0.0191 (5)	0.0267 (5)	0.0213 (5)	−0.0009 (4)	0.0023 (4)	0.0007 (4)
C9	0.0309 (6)	0.0304 (6)	0.0322 (6)	0.0033 (5)	0.0013 (5)	−0.0073 (5)
C10	0.0323 (7)	0.0230 (6)	0.0488 (8)	−0.0019 (5)	−0.0025 (6)	−0.0020 (5)
C11	0.0230 (6)	0.0291 (6)	0.0439 (7)	−0.0021 (5)	0.0036 (5)	0.0120 (6)
N3	0.0168 (5)	0.0310 (5)	0.0289 (5)	−0.0009 (4)	0.0052 (4)	0.0061 (4)
C12	0.0157 (5)	0.0292 (6)	0.0226 (5)	0.0007 (4)	0.0018 (4)	−0.0027 (5)
C13	0.0329 (7)	0.0418 (7)	0.0331 (7)	0.0005 (6)	0.0084 (5)	−0.0108 (6)
N4	0.0139 (4)	0.0379 (6)	0.0275 (5)	−0.0007 (4)	0.0030 (4)	−0.0043 (4)
C14	0.0268 (6)	0.0429 (8)	0.0298 (6)	−0.0083 (5)	0.0009 (5)	−0.0097 (5)
C15	0.0377 (7)	0.0336 (7)	0.0498 (8)	−0.0065 (6)	0.0080 (6)	−0.0106 (6)
C16	0.0289 (6)	0.0289 (7)	0.0483 (8)	0.0030 (5)	0.0089 (6)	0.0010 (6)
N5	0.0191 (5)	0.0340 (6)	0.0264 (5)	−0.0008 (4)	0.0012 (4)	0.0034 (4)
N1'	0.0170 (4)	0.0236 (5)	0.0187 (4)	0.0017 (3)	0.0028 (3)	0.0017 (4)
C2'	0.0189 (5)	0.0215 (5)	0.0202 (5)	0.0008 (4)	0.0023 (4)	0.0024 (4)
C3'	0.0245 (6)	0.0293 (6)	0.0207 (6)	0.0008 (5)	0.0036 (4)	0.0076 (5)
C4'	0.0267 (6)	0.0338 (6)	0.0208 (6)	−0.0018 (5)	0.0093 (4)	0.0047 (5)
C5'	0.0180 (5)	0.0328 (6)	0.0247 (6)	0.0012 (4)	0.0066 (4)	0.0021 (5)
C6'	0.0173 (5)	0.0216 (5)	0.0208 (5)	0.0003 (4)	0.0033 (4)	0.0003 (4)
C7'	0.0189 (5)	0.0275 (6)	0.0189 (5)	0.0041 (4)	0.0025 (4)	0.0033 (4)
C8'	0.0259 (6)	0.0290 (6)	0.0351 (7)	0.0041 (5)	0.0054 (5)	0.0001 (5)
N2'	0.0222 (5)	0.0360 (6)	0.0212 (5)	0.0102 (4)	−0.0005 (4)	0.0057 (4)
C9'	0.0226 (6)	0.0433 (7)	0.0183 (5)	0.0057 (5)	−0.0005 (4)	−0.0012 (5)
C10'	0.0198 (5)	0.0392 (7)	0.0276 (6)	−0.0005 (5)	0.0032 (5)	−0.0072 (5)
C11'	0.0210 (5)	0.0284 (6)	0.0254 (6)	0.0007 (4)	0.0057 (4)	0.0016 (5)
N3'	0.0188 (4)	0.0295 (5)	0.0173 (5)	0.0020 (4)	0.0023 (4)	0.0013 (4)
C12'	0.0168 (5)	0.0264 (6)	0.0192 (5)	0.0033 (4)	0.0035 (4)	0.0016 (4)
C13'	0.0319 (6)	0.0324 (7)	0.0306 (6)	0.0023 (5)	−0.0060 (5)	−0.0019 (5)
N4'	0.0179 (5)	0.0331 (5)	0.0249 (5)	0.0073 (4)	0.0026 (4)	0.0025 (4)
C14'	0.0372 (7)	0.0330 (7)	0.0345 (7)	0.0135 (5)	0.0000 (6)	−0.0044 (5)
C15'	0.0383 (7)	0.0274 (7)	0.0484 (8)	−0.0012 (5)	−0.0094 (6)	0.0076 (6)
C16'	0.0224 (6)	0.0410 (7)	0.0421 (8)	−0.0046 (5)	−0.0018 (5)	0.0194 (6)
N5'	0.0213 (5)	0.0400 (6)	0.0242 (5)	0.0072 (4)	0.0074 (4)	0.0091 (4)

### Geometric parameters (Å, °)

**Table:**

N1—C2	1.3471 (15)	N1'—C2'	1.3456 (14)
N1—C6	1.3494 (14)	N1'—C6'	1.3523 (14)
C2—C3	1.3908 (17)	C2'—C3'	1.3992 (16)
C2—C7	1.5504 (15)	C2'—C7'	1.5515 (15)
C3—C4	1.3943 (18)	C3'—C4'	1.3909 (16)
C3—H3	0.9500	C3'—H3'	0.9500
C4—C5	1.3903 (19)	C4'—C5'	1.3892 (17)
C4—H4	0.9500	C4'—H4'	0.9500
C5—C6	1.3949 (17)	C5'—C6'	1.3916 (16)
C5—H5	0.9500	C5'—H5'	0.9500
C6—C12	1.5453 (16)	C6'—C12'	1.5499 (15)
C7—N3	1.4709 (14)	C7'—N2'	1.4742 (15)
C7—N2	1.4786 (15)	C7'—N3'	1.4752 (15)
C7—C8	1.5297 (16)	C7'—C8'	1.5337 (17)
C8—H8A	0.9800	C8'—H8'1	0.9800
C8—H8B	0.9800	C8'—H8'2	0.9800
C8—H8C	0.9800	C8'—H8'3	0.9800
N2—C9	1.4676 (16)	N2'—C9'	1.4774 (17)
N2—H2N	0.893 (16)	N2'—H2'N	0.887 (15)
C9—C10	1.5217 (19)	C9'—C10'	1.5284 (18)
C9—H9A	0.9900	C9'—H9'1	0.9900
C9—H9B	0.9900	C9'—H9'2	0.9900
C10—C11	1.527 (2)	C10'—C11'	1.5297 (17)
C10—H10A	0.9900	C10'—H10C	0.9900
C10—H10B	0.9900	C10'—H10D	0.9900
C11—N3	1.4758 (17)	C11'—N3'	1.4707 (15)
C11—H11A	0.9900	C11'—H11C	0.9900
C11—H11B	0.9900	C11'—H11D	0.9900
N3—H3N	0.894 (14)	N3'—H3'N	0.873 (15)
C12—N4	1.4653 (14)	C12'—N4'	1.4676 (14)
C12—N5	1.4862 (15)	C12'—N5'	1.4830 (15)
C12—C13	1.5293 (17)	C12'—C13'	1.5318 (16)
C13—H13A	0.9800	C13'—H13D	0.9800
C13—H13B	0.9800	C13'—H13E	0.9800
C13—H13C	0.9800	C13'—H13F	0.9800
N4—C14	1.4683 (16)	N4'—C14'	1.4715 (17)
N4—H4N	0.908 (16)	N4'—H4'N	0.894 (14)
C14—C15	1.523 (2)	C14'—C15'	1.524 (2)
C14—H14A	0.9900	C14'—H14C	0.9900
C14—H14B	0.9900	C14'—H14D	0.9900
C15—C16	1.5271 (19)	C15'—C16'	1.522 (2)
C15—H15A	0.9900	C15'—H15C	0.9900
C15—H15B	0.9900	C15'—H15D	0.9900
C16—N5	1.4764 (17)	C16'—N5'	1.4715 (18)
C16—H16A	0.9900	C16'—H16C	0.9900
C16—H16B	0.9900	C16'—H16D	0.9900
N5—H5N	0.901 (15)	N5'—H5'N	0.905 (17)
			
C2—N1—C6	119.31 (9)	C2'—N1'—C6'	118.98 (9)
N1—C2—C3	122.14 (10)	N1'—C2'—C3'	122.03 (10)
N1—C2—C7	114.37 (9)	N1'—C2'—C7'	115.63 (9)
C3—C2—C7	123.29 (10)	C3'—C2'—C7'	122.28 (10)
C2—C3—C4	118.36 (12)	C4'—C3'—C2'	118.40 (10)
C2—C3—H3	120.8	C4'—C3'—H3'	120.8
C4—C3—H3	120.8	C2'—C3'—H3'	120.8
C5—C4—C3	119.79 (11)	C5'—C4'—C3'	119.90 (11)
C5—C4—H4	120.1	C5'—C4'—H4'	120.1
C3—C4—H4	120.1	C3'—C4'—H4'	120.1
C4—C5—C6	118.44 (11)	C4'—C5'—C6'	118.32 (10)
C4—C5—H5	120.8	C4'—C5'—H5'	120.8
C6—C5—H5	120.8	C6'—C5'—H5'	120.8
N1—C6—C5	121.89 (11)	N1'—C6'—C5'	122.36 (10)
N1—C6—C12	114.35 (9)	N1'—C6'—C12'	115.19 (9)
C5—C6—C12	123.73 (10)	C5'—C6'—C12'	122.38 (9)
N3—C7—N2	111.31 (9)	N2'—C7'—N3'	110.01 (9)
N3—C7—C8	108.63 (9)	N2'—C7'—C8'	109.07 (9)
N2—C7—C8	107.05 (9)	N3'—C7'—C8'	107.73 (9)
N3—C7—C2	109.88 (9)	N2'—C7'—C2'	108.77 (9)
N2—C7—C2	112.93 (9)	N3'—C7'—C2'	114.13 (9)
C8—C7—C2	106.82 (9)	C8'—C7'—C2'	106.99 (9)
C7—C8—H8A	109.5	C7'—C8'—H8'1	109.5
C7—C8—H8B	109.5	C7'—C8'—H8'2	109.5
H8A—C8—H8B	109.5	H8'1—C8'—H8'2	109.5
C7—C8—H8C	109.5	C7'—C8'—H8'3	109.5
H8A—C8—H8C	109.5	H8'1—C8'—H8'3	109.5
H8B—C8—H8C	109.5	H8'2—C8'—H8'3	109.5
C9—N2—C7	112.79 (9)	C7'—N2'—C9'	112.56 (9)
C9—N2—H2N	108.2 (9)	C7'—N2'—H2'N	106.5 (9)
C7—N2—H2N	109.7 (9)	C9'—N2'—H2'N	108.0 (9)
N2—C9—C10	108.94 (10)	N2'—C9'—C10'	113.59 (10)
N2—C9—H9A	109.9	N2'—C9'—H9'1	108.8
C10—C9—H9A	109.9	C10'—C9'—H9'1	108.8
N2—C9—H9B	109.9	N2'—C9'—H9'2	108.8
C10—C9—H9B	109.9	C10'—C9'—H9'2	108.8
H9A—C9—H9B	108.3	H9'1—C9'—H9'2	107.7
C9—C10—C11	108.86 (10)	C9'—C10'—C11'	109.20 (9)
C9—C10—H10A	109.9	C9'—C10'—H10C	109.8
C11—C10—H10A	109.9	C11'—C10'—H10C	109.8
C9—C10—H10B	109.9	C9'—C10'—H10D	109.8
C11—C10—H10B	109.9	C11'—C10'—H10D	109.8
H10A—C10—H10B	108.3	H10C—C10'—H10D	108.3
N3—C11—C10	112.98 (10)	N3'—C11'—C10'	108.81 (9)
N3—C11—H11A	109.0	N3'—C11'—H11C	109.9
C10—C11—H11A	109.0	C10'—C11'—H11C	109.9
N3—C11—H11B	109.0	N3'—C11'—H11D	109.9
C10—C11—H11B	109.0	C10'—C11'—H11D	109.9
H11A—C11—H11B	107.8	H11C—C11'—H11D	108.3
C7—N3—C11	112.97 (10)	C11'—N3'—C7'	112.85 (9)
C7—N3—H3N	106.7 (9)	C11'—N3'—H3'N	111.1 (9)
C11—N3—H3N	107.8 (9)	C7'—N3'—H3'N	109.3 (9)
N4—C12—N5	111.38 (9)	N4'—C12'—N5'	111.85 (9)
N4—C12—C13	108.09 (9)	N4'—C12'—C13'	107.93 (9)
N5—C12—C13	107.68 (10)	N5'—C12'—C13'	107.19 (9)
N4—C12—C6	110.20 (9)	N4'—C12'—C6'	110.29 (9)
N5—C12—C6	111.44 (9)	N5'—C12'—C6'	111.41 (8)
C13—C12—C6	107.89 (10)	C13'—C12'—C6'	107.99 (9)
C12—C13—H13A	109.5	C12'—C13'—H13D	109.5
C12—C13—H13B	109.5	C12'—C13'—H13E	109.5
H13A—C13—H13B	109.5	H13D—C13'—H13E	109.5
C12—C13—H13C	109.5	C12'—C13'—H13F	109.5
H13A—C13—H13C	109.5	H13D—C13'—H13F	109.5
H13B—C13—H13C	109.5	H13E—C13'—H13F	109.5
C12—N4—C14	113.55 (9)	C12'—N4'—C14'	112.71 (9)
C12—N4—H4N	106.1 (10)	C12'—N4'—H4'N	107.1 (9)
C14—N4—H4N	109.3 (10)	C14'—N4'—H4'N	109.8 (9)
N4—C14—C15	113.43 (11)	N4'—C14'—C15'	113.76 (11)
N4—C14—H14A	108.9	N4'—C14'—H14C	108.8
C15—C14—H14A	108.9	C15'—C14'—H14C	108.8
N4—C14—H14B	108.9	N4'—C14'—H14D	108.8
C15—C14—H14B	108.9	C15'—C14'—H14D	108.8
H14A—C14—H14B	107.7	H14C—C14'—H14D	107.7
C14—C15—C16	109.68 (11)	C16'—C15'—C14'	109.76 (11)
C14—C15—H15A	109.7	C16'—C15'—H15C	109.7
C16—C15—H15A	109.7	C14'—C15'—H15C	109.7
C14—C15—H15B	109.7	C16'—C15'—H15D	109.7
C16—C15—H15B	109.7	C14'—C15'—H15D	109.7
H15A—C15—H15B	108.2	H15C—C15'—H15D	108.2
N5—C16—C15	109.18 (10)	N5'—C16'—C15'	109.26 (10)
N5—C16—H16A	109.8	N5'—C16'—H16C	109.8
C15—C16—H16A	109.8	C15'—C16'—H16C	109.8
N5—C16—H16B	109.8	N5'—C16'—H16D	109.8
C15—C16—H16B	109.8	C15'—C16'—H16D	109.8
H16A—C16—H16B	108.3	H16C—C16'—H16D	108.3
C16—N5—C12	112.33 (9)	C16'—N5'—C12'	112.71 (10)
C16—N5—H5N	106.5 (9)	C16'—N5'—H5'N	106.6 (10)
C12—N5—H5N	106.9 (9)	C12'—N5'—H5'N	103.9 (10)
			
C6—N1—C2—C3	−0.52 (17)	C6'—N1'—C2'—C3'	−1.46 (16)
C6—N1—C2—C7	−175.43 (9)	C6'—N1'—C2'—C7'	−178.67 (9)
N1—C2—C3—C4	2.3 (2)	N1'—C2'—C3'—C4'	1.42 (17)
C7—C2—C3—C4	176.79 (12)	C7'—C2'—C3'—C4'	178.44 (10)
C2—C3—C4—C5	−1.6 (2)	C2'—C3'—C4'—C5'	−0.30 (18)
C3—C4—C5—C6	−0.8 (2)	C3'—C4'—C5'—C6'	−0.70 (18)
C2—N1—C6—C5	−2.05 (17)	C2'—N1'—C6'—C5'	0.39 (16)
C2—N1—C6—C12	179.94 (9)	C2'—N1'—C6'—C12'	177.37 (9)
C4—C5—C6—N1	2.68 (19)	C4'—C5'—C6'—N1'	0.68 (17)
C4—C5—C6—C12	−179.50 (12)	C4'—C5'—C6'—C12'	−176.09 (10)
N1—C2—C7—N3	−169.79 (9)	N1'—C2'—C7'—N2'	−159.75 (9)
C3—C2—C7—N3	15.36 (15)	C3'—C2'—C7'—N2'	23.05 (14)
N1—C2—C7—N2	−44.86 (13)	N1'—C2'—C7'—N3'	−36.50 (13)
C3—C2—C7—N2	140.29 (12)	C3'—C2'—C7'—N3'	146.30 (11)
N1—C2—C7—C8	72.55 (12)	N1'—C2'—C7'—C8'	82.56 (12)
C3—C2—C7—C8	−102.30 (13)	C3'—C2'—C7'—C8'	−94.63 (12)
N3—C7—N2—C9	56.72 (12)	N3'—C7'—N2'—C9'	−53.26 (12)
C8—C7—N2—C9	175.30 (9)	C8'—C7'—N2'—C9'	−171.21 (10)
C2—C7—N2—C9	−67.42 (12)	C2'—C7'—N2'—C9'	72.43 (12)
C7—N2—C9—C10	−60.40 (12)	C7'—N2'—C9'—C10'	51.37 (13)
N2—C9—C10—C11	57.28 (13)	N2'—C9'—C10'—C11'	−51.67 (13)
C9—C10—C11—N3	−53.72 (13)	C9'—C10'—C11'—N3'	55.04 (12)
N2—C7—N3—C11	−50.79 (12)	C10'—C11'—N3'—C7'	−61.18 (11)
C8—C7—N3—C11	−168.41 (9)	N2'—C7'—N3'—C11'	59.80 (11)
C2—C7—N3—C11	75.06 (11)	C8'—C7'—N3'—C11'	178.57 (9)
C10—C11—N3—C7	50.98 (13)	C2'—C7'—N3'—C11'	−62.78 (12)
N1—C6—C12—N4	−161.87 (9)	N1'—C6'—C12'—N4'	151.51 (9)
C5—C6—C12—N4	20.16 (16)	C5'—C6'—C12'—N4'	−31.51 (15)
N1—C6—C12—N5	−37.69 (13)	N1'—C6'—C12'—N5'	26.68 (13)
C5—C6—C12—N5	144.34 (12)	C5'—C6'—C12'—N5'	−156.34 (10)
N1—C6—C12—C13	80.32 (12)	N1'—C6'—C12'—C13'	−90.77 (11)
C5—C6—C12—C13	−97.65 (13)	C5'—C6'—C12'—C13'	86.21 (13)
N5—C12—N4—C14	−51.19 (13)	N5'—C12'—N4'—C14'	50.94 (13)
C13—C12—N4—C14	−169.29 (11)	C13'—C12'—N4'—C14'	168.61 (10)
C6—C12—N4—C14	73.03 (13)	C6'—C12'—N4'—C14'	−73.64 (12)
C12—N4—C14—C15	50.09 (15)	C12'—N4'—C14'—C15'	−50.04 (13)
N4—C14—C15—C16	−51.80 (15)	N4'—C14'—C15'—C16'	52.01 (14)
C14—C15—C16—N5	55.64 (15)	C14'—C15'—C16'—N5'	−55.21 (14)
C15—C16—N5—C12	−59.44 (14)	C15'—C16'—N5'—C12'	58.90 (13)
N4—C12—N5—C16	56.92 (12)	N4'—C12'—N5'—C16'	−56.78 (12)
C13—C12—N5—C16	175.27 (9)	C13'—C12'—N5'—C16'	−174.89 (9)
C6—C12—N5—C16	−66.59 (12)	C6'—C12'—N5'—C16'	67.17 (12)

### Hydrogen-bond geometry (Å, °)

**Table:**

*D*—H···*A*	*D*—H	H···*A*	*D*···*A*	*D*—H···*A*
N2—H2N···N5^i^	0.893 (16)	2.596 (15)	3.3984 (16)	149.9 (12)
N4—H4N···N4'^ii^	0.908 (16)	2.623 (16)	3.4716 (16)	155.8 (13)
N3'—H3'N···N5'^iii^	0.873 (15)	2.418 (15)	3.2662 (17)	164.2 (12)

Symmetry codes: (i) −*x*, −*y*+1, −*z*+1; (ii) *x*−1, *y*, *z*; (iii) −*x*+1, −*y*+1, −*z*+1.
